# Age-related differences in information, but not task control in the color-word Stroop task

**DOI:** 10.3758/s13423-024-02631-z

**Published:** 2025-01-17

**Authors:** Eldad Keha, Daniela Aisenberg-Shafran, Shachar Hochman, Eyal Kalanthroff

**Affiliations:** 1 Department of Psychology, The Hebrew University of Jerusalem, Mt. Scopus, 91905 Jerusalem, Israel; 2 Department of Psychology, Achva Academic College, Arugot, Israel; 3 Department of Clinical Psychology of Adulthood and Aging, Ruppin Academic Center, Emek Hefer, Israel; 4 School of Psychology, University of Surrey, Guildford, GU2 7XH UK

**Keywords:** Older adults, Stroop task, Task conflict, Task control, Information control

## Abstract

Older adults were found to struggle with tasks that require cognitive control. One task that measures the ability to exert cognitive control is the color-word Stroop task. Almost all studies that tested cognitive control in older adults using the Stroop task have focused on one type of control – Information control. In the present work, we ask whether older adults also show a deficit in another type of cognitive control – Task control. To that end, we tested older and younger adults by isolating and measuring two types of conflict – information conflict and task conflict. Information conflict was measured by the difference between color identification of incongruent color words and color identification of neutral words, while task conflict was measured by the difference between color identification of neutral words and color identification of neutral symbols and by the reverse facilitation effect. We tested how the behavioral markers of these two types of conflicts are affected under low task control conditions, which is essential for measuring task conflict behaviorally. Older adults demonstrated a deficit in information control by showing a larger information conflict marker, but not in task control markers, as no differences in task conflict were found between younger and older adults. These findings supported previous studies that work against theories that link the larger Stroop interference in older adults to a generic slowdown or a generic inhibitory failure. We discussed the relevancy of the results and future research directions in line with other Stroop studies that tested age-related differences in different control mechanisms.

**Supplementary Information:**

The online version contains supplementary material available at 10.3758/s13423-024-02631-z.

## Introduction

The Stroop task (Stroop, 1935) is a standard paradigm for measuring cognitive control (Egner & Hirsch, 2005) in which participants are required to identify the color of written words and to ignore the irrelevant dimension of reading the words. The words can be incongruent with the color of the stimulus (e.g., the word RED appears in blue), congruent (e.g., RED appears in red), or neutral (e.g., TABLE or the same-letter string "XXXX" appears in red). Recent research has suggested that the Stroop task can be broken down into two main conflicts (Kalanthroff et al., 2018): The first, *information conflict*, represents the interference from the semantic processing of word meaning and arises in incongruent conditions. The second, *task conflict*, represents the interference from the activation of the competing reading task and arises not only in incongruent trials but also in other conditions in which participants engage in reading, including congruent and word-neutral trials (see Kalanthroff et al., 2018; Littman et al., 2019; Parris et al., 2022). The information conflict is commonly assessed by the difference in reaction time (RT) or error rate between incongruent and neutral conditions, referred to as the “interference effect.” In contrast, task conflict is often measured by the difference in RT or error rate between neutral same-letter strings (e.g., "XXXX" in red) and congruent conditions, referred to as the “facilitation effect” (Goldfarb & Henik, 2007; Kalanthroff et al., 2013; Keha & Kalanthroff, 2023b). Alternatively, task conflict can also be measured by the difference in RT or error rate between neutral words and same-letter strings (Augustinova et al., 2019), or between neutral words and symbols strings, referred to as the “word interference effect” (Keha & Kalanthroff, 2023a; Kinoshita et al., 2018; Mills, 2017). In recent years, these markers for task and information conflict have been widely used to distinguish between different types of conflicts in various studies, including behavioral (Augustinova et al., 2019; Entel et al., 2015; Keha & Kalanthroff, 2023a, 2023b; Shichel & Tzelgov, 2018), pupillometry (Hasshim & Parris, 2015), computational modeling (Kalanthroff et al., 2018), and neuronal research (Parris et al., 2019, 2021).

It is important to mention that common results in the Stroop task indicate faster RTs to congruent compared to same-letter strings neutral trials, a finding which seemingly contradicts the notion that congruent Stroop trials trigger a conflict. However, studies have shown that this contradiction can be settled by considering the existence of a very efficient proactive task control mechanism (Goldfarb & Henik, 2007; Kalanthroff & Henik, 2014; Kalanthroff et al., 2018; Littman et al., 2019). Indeed, under reduced levels of task control, the behavioral manifestation of task conflict becomes apparent. Reduced levels of task control can arise from either experimental manipulation (Keha & Kalanthroff, 2023a) or inherent trait characteristics (Kalanthroff et al., 2016). One commonly used experimental manipulation that effectively lowers (relaxes) levels of task control and produces a strong behavioral manifestation of task conflict (e.g., slower RTs for congruent vs. same-letter strings neutral trials, known as the *reverse facilitation* effect) is the "proportion of non-word trials” manipulation, which involves presenting a majority of neutral non-words trials within a block (Goldfarb & Henik, 2007; Keha & Kalanthroff, 2023a; Parris, 2014). With respect to inherent trait characteristics, several studies have identified reduced levels of task control in different clinical populations. For instance, individuals with obsessive–compulsive disorder (OCD) have demonstrated less efficient task control when faced with both information and task conflicts (Kalanthroff et al., 2017; Marsh et al., 2014). Furthermore, reduced task control abilities have been observed in young children, as they exhibit longer RTs when asked to identify the color of colored objects compared to colored abstract shapes (La Heij & Boelens, 2011; La Heij et al., 2010). Although extensive literature exists on task control in various populations, one particular group has been overlooked in this research: older adults. The objective of the present study was to address this gap in the literature by examining task and information control in older adults.

Although no attention has been given to task control in older adults, studies have been conducted on other aspects of cognitive control in this population. Most studies have shown that older adults exhibit a general difficulty in cognitive control which reduces their performance in tasks that require selective attention, sustained attention, and various executive functions (Braver & Barch, 2002; Braver et al., 2021). The most dominant model to account for these findings is the *inhibitory deficit* theory, which suggests a general deficit in inhibition in older adults. This theory is supported by both behavioral studies, which show that older adults are more influenced by irrelevant or distracting information (Chiappe et al., 2000; Connelly et al., 1991; Duchek et al., 1995; Sommers & Danielson, 1999) and by neuroimaging studies, which show that while conducting a task that requires executive functions, older adults struggle in inhibitory processes and exhibit a stronger related activity in the inferior frontal gyrus, a brain area which is highly involved in inhibitory processes (Langenecker et al., 2004; Zysset et al., 2007). Importantly, many of the behavioral and brain imaging studies on which the inhibitory deficit theory is based have used the Stroop task. Specifically, studies that used this task consistently find a greater interference effect for older compared to younger adults, which signifies a cognitive control deficit (Bugg et al., 2007; Cohn et al., 1984; Verhaeghen & De Meersman, 1998; West & Alain, 2000; Wolf et al., 2014).

It is important to note that despite this comprehensive evidence, almost all previous studies that tested age differences in cognitive control using the Stroop task focused solely on information conflict and neglected other conflict markers. In other words, all these studies overlooked the important mechanism of task control. Consistent with the inhibitory deficit theory, these studies have consistently shown a larger interference effect (i.e., less efficient information control) in older compared to younger adults (e.g., Augustinova et al., 2018; Burca et al., 2022; Cohn et al., 1984; Davidson et al., 2003; Li & Bosman, 1996; Nicosia & Balota, 2020; Spieler et al., 1996; Verhaegen & De Meersman, 1998; West & Alain, 2000). Task control, while being mostly dissociable and independent from information control (Steinhauser & Hübner, 2009), has also been shown to be highly contingent upon inhibitory control (Kalanthroff et al., 2013). Thus, according to the inhibition deficit theory, it is expected that task control will also be deficient in older adults. However, although task control was not tested in older adults, one early study presented surprising data that might be relevant to the current investigation. Li and Bosman (1996) administered a version of the Stroop task to older and younger adults and used both word neutrals and non-word neutrals (e.g., ****). First, consistent with other studies, these researchers found a significantly larger interference effect in older compared to younger adults. However, their data suggests no difference between the word and non-word neutral conditions. Given that the difference between these two conditions is a reliable measure for task control, this surprising data suggests that there is no difference in task control between older and younger adults – a finding that might contradict the classic inhibition deficit theory. If correct, this suggests that a more accurate theory is warranted. For example, while it might be harder for older adults to inhibit irrelevant distracting information, their ability to inhibit irrelevant stimulus-driven behaviors may remain intact. This dissociation might suggest that the global inhibition deficit theory needs to be redefined. Furthermore, as task control has been associated with abnormal behavioral phenomena (e.g., compulsive behaviors; Kalanthroff et al., 2017), such a dissociation might have a crucial implication for our understanding of the specific cognitive deficit in older adults.

The current study aimed to investigate information and task control in the Stroop task in older and younger adults. To that aim, we administered the Stroop task with four different stimulus-type conditions (congruent, neutral words, neutral symbols, and incongruent conditions). To test information control, we compared neutral words and incongruent trials. To test task control, we used two common markers for task conflict – the reverse facilitation effect (congruent vs. symbols; Kalanthroff et al., 2018) and the word interference effect (neutral word vs. neutral symbols; Kinoshita et al., 2018). To be able to observe task conflict, we used the proportion of non-word trials manipulation to reduce task control levels by lowering the proportion of conflict-laden trials (Kalanthroff et al., 2018; Tzelgov et al., 1992). Therefore, we compared both information and task conflict in older and younger adults, under low control (67% neutral symbols) conditions. Based on the studies reviewed above, we expected to find reduced information control in older adults as compared to younger adults, which will be indicated by a larger interference effect. With respect to task control, the literature is mixed, thus it is harder to draw clear hypotheses. The inhibition deficit theory suggests reduced task control in older adults. However, Li and Bosman's (1996) initial data suggest comparable task control in older and younger adults. Hence, we investigated this important question at an exploratory level.

## Method

### Participants

Fifty-eight participants, 29 older adults and 29 younger adults, participated in the experiment. Older adults were between the ages of 65 and 86 (M = 73.07, SD = 5.23) years, while younger adults were between the ages of 18 and 29 (M = 23.70, SD = 2.49) years. The participants in the younger adults group were college students and were rewarded for their participation with course credit or small monetary compensation (~ 5 USD). The older adults group consisted of participants recruited from a local community in the same area of the college. As a token of appreciation, a lecture in the community was given to the older adults group by one of the authors (DA). All participants were native Hebrew speakers and had normal or corrected-to-normal vision. For participants in the older adults group, a score lower than 24 on the Mini-Mental State Examination (Folstein et al., 1983) was an exclusion criterion (one participant was excluded due to this criterion). In addition, three participants in the younger adults group were excluded from the analysis due to extremely low overall accuracy rates in the Stroop task (over 3 SDs below the mean accuracy of the corresponding age group), which indicates either deficient cognitive abilities or low task engagement. The analyzed sample therefore included 28 older adults and 26 younger adults. Sample characteristics are presented in Table 1. The data of both older and younger adults was collected between May 2020 and March 2021.

The sample size was determined based on a conflict type × age group interaction that was reported by Augustinova et al. (2018) with a ${\eta }_{p}^{2}$ effect size of 0.063. We ran an a priori analysis to compute the required sample size with G-power (Faul et al., 2007). To achieve a power of 80% with two age groups and four measurements (stimulus type conditions) with a Type I error of 0.05, the total required sample size was 58 participants.

**Table 1:** Sample characteristics by age groups

Age group	Older adults (*N* = 28)	Younger adults (*N* = 27)
Male/female Right/left	11/17 24/4	23/4 24/3
Age, years	73.07 (5.23) [65–86]	23.70 (2.49) [18–29]
Education, years	14.21 (2.60) [10–20]	12.29 (1.10) [12–17]
Mini-Mental State Examination score	27.85 (0.6) [27–29]	

Values are given as mean (SDs) [range], for all the demographic and clinical variables

### Procedure

The experiment was approved by the Hebrew University Ethical Committee (HUJI-2021–06011) and all participants signed an informed consent form prior to their participation in the study. The older adults group first performed the Mini-Mental State Examination. Next, both groups completed the low-control Stroop task. At the end of the experiment, participants were thanked and debriefed.

### The Stroop task

Participants sat approximately 60 cm from the computer screen and were instructed to place their dominant hand on the C, V, B, and N keys and respond as quickly and accurately as possible to the ink color of the stimuli. Colored stickers of red, blue, green, and yellow were placed on the keyboard as indicators for the correct color-key mapping. Response keys were counterbalanced between participants. First, participants completed a practice block that consisted of 48 trials of key-response mapping training in which they were instructed to respond to the color of an asterisk at the center of the screen. Participants received feedback for mistakes or for failing to respond in 2,000 ms. Next, participants started the experimental block and were reminded to respond as quickly and accurately as possible to the ink color of the stimuli and to ignore the written words. All of the stimuli in the experiment were presented in one of four possible ink colors (red, green, blue, or yellow) and were divided into four different categories of four possible stimuli each^1^: (1) Neutral symbols – a string of four different symbols (+ = !/, *?!#, ~ > !", %$!@); (2) Neutral words – three- or four-letter words in Hebrew (form, pot, stall, oven) selected from a word-frequency database (Frost & Plaut, 2005); (3) Incongruent – color words that do not match the color of the word (e.g., YELLOW presented in red color); and (4) Congruent – color words that match the color of the word (e.g., RED presented in red color). To reduce the threat of response contingency learning (Schmidt, 2019), the number of stimuli in each condition was identical, such that each stimulus in each condition was presented in just one out of the four possible colors. Stimuli for the experiment were presented at the center of the screen on a black background in bold Arial font (22 points). The experiment was administered using E-prime 3.0.3.80 psychology software. The experimental block consisted of 288 trials comprising 192 neutral symbols, 32 neutral words, 32 congruent, and 32 incongruent. Each trial began with a 1,000-ms fixation point that was followed by the Stroop stimuli that appeared for 2,000 ms or until response. Each trial ended with a 500-ms black screen interval.

### Statistical analysis

**Fig. 1:**
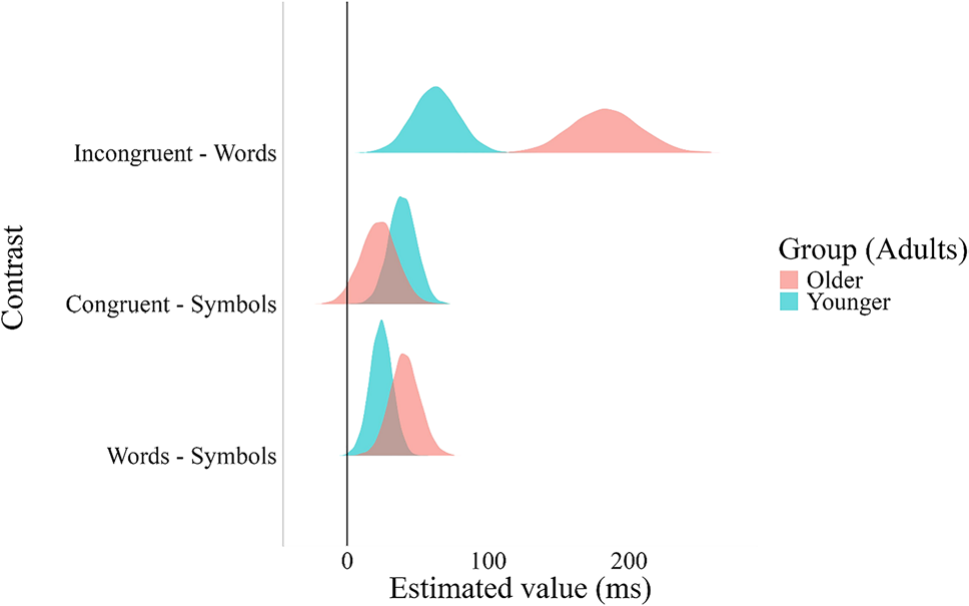
Posterior distributions of the Stroop contrasts by age group. Information conflict is measured by the difference between incongruent and neutral words, while task conflict is measured by both the word interference effect task conflict measure (neutral words vs. neutral symbols) and the reverse facilitation effect (congruent vs. neutral symbols). The degree of overlap between the distributions indicates the differences between the groups, with little overlap suggesting a large difference between the groups

We fit a Bayesian log-normal linear model to predict Stroop RT, which included one between-participant variable: group (older adults vs. younger adults), one within-participant variable: stimulus type (neutral symbols, neutral words, incongruent, and congruent), and the interaction effect between these two predictors. We used conservative priors for the coefficients with normal distributions centered on zero, and different standard deviations (SDs) for the different effects (a full summary and specification of the model are given in Appendix A). To address the concern of generalized slowing in reaction time (RT) analyses (Faust et al., 1999), which raises the concern that slower processing speed in older adults accounts for the larger interference in that group, we applied in our model a log-normal transformation of the RT distribution. This transformation controls for differences in processing speed both between and within groups by normalizing the RTs, thereby allowing for a more accurate assessment of the underlying processes. By taking this approach, we reduced biases associated with group differences in general RTs, ensuring that our findings were not confounded by variations in response speed. For this analysis, only correct trials that were within 3 SDs of the mean were analyzed (for the same participants within the same condition). Less than 1% of the trials were excluded. We used Markov Chain Monte Carlo (MCMC) sampling with four chains of 8,000 iterations and a warmup of 4,000 iterations for the model. The explained variance of the model was 49% (R^2^ = 0.492), indicating high explanatory power (see predicted values in Fig. 1). Inference regarding the effects was conducted by inspecting the 95% highest density interval (HDI) of the posterior distribution of the effect of interest. If the null value (i.e., zero) was not included in the interval, this indicated that there is a 95% chance or more that the effect exists, and the null hypothesis can be rejected (Kruschke, 2014). The model was fit using the *brms* package in R (Bürkner, 2017), and further analyses were conducted using the *bayestestR* (Makowski et al., 2019) package. The exclusion of participants and trials from the analysis was determined based on our previous analysis using the same paradigm (see Keha & Kalanthroff, 2023a).

In addition to the Bayesian analysis and the log-normal transformation of RTs, we have also carried out a traditional two-way mixed-model analysis of variance (ANOVA) for the RT z-scores (following the method by Jackson & Balota, 2013), with stimulus-type as the within-subject variable and group as the between-subject variable (see Online Supplementary Material (OSM) B).

## Results

Based on our hypotheses, we tested information and task conflicts separately. Our results indicate that RTs for the incongruent condition were longer compared to the neutral words condition (median = 123.1, HDI [150.2, 96.2]; Table 2). Information conflict (incongruent RT minus neutral word RT) in the older adults group was larger as compared with information conflict in the younger adults group (median = 120.0, HDI [58.1, 180.7]; see Table 2 and Fig. 1). For the word interference effect measure, our results indicated that the RTs for the neutral words condition were longer compared to the neutral symbols condition (median = 32.2, HDI [20.3, 45.5]). However, this effect did not differ between older and younger adults (median = 16.8, HDI [−10.1, 44.2]). Finally, for the other task conflict measure – the reverse facilitation effect (congruent RT vs. neutral symbols RT), we found that the neutral symbols condition was faster compared to the congruent condition (median = 30.8, HDI [16.2, 46.1]). As in the word interference effect measure, the reverse facilitation effect did not differ between older and younger adults (median = −16.8, HDI [−49.6, 17.1]).^2^

**Table 2:** Descriptive results for the Stroop task

	Congruent	Neutral words	Incongruent	Neutral symbols	Group mean
Older adults	927 (120) [98.73]	936 (116) [98.97]	1133 (174) [90.95]	902 (117) [98.50]	974 (161) [96.79]
Younger adults	663 (99) [94.29]	651 (82) [93.87]	723 (127) [92.03]	625 (92) [90.9]	662 (94) [93.40]
Condition mean	803 (184) [96.93]	803 (189) [96.95]	879 (236) [93.33]	789 (181) [96.90]	

Values are given as means (SD) [accuracy] data of each condition in each group

## Discussion

In the present study, we investigated whether older adults exhibit a deficit in task control in the Stroop task. To that aim, we used a low proportion of conflict trials to reduce task control levels by lowering the proportion of conflict-laden trials. First, we replicated previous findings showing an age-related deficit in information control by showing a larger difference between incongruent and neutral words trials in older adults (Augustinova et al., 2018; Bugg et al., 2007; Burca et al., 2022; Cohn et al., 1984; Verhaeghen & De Meersman, 1998; West & Alain, 2000; Wolf et al., 2014). Interestingly, there were no differences between the groups in either the difference between congruent and neutral symbols trials or between neutral words and neutral symbols trials, which indicated no deficit in task control in older adults. This pattern of results supports a dissociation between information and task control and suggests that the deficit in cognitive control often seen in older adults is not global and remains focused on specific functioning.

In line with previous studies (Bugg et al., 2006, 2007; Cohn et al., 1984; Wolf et al., 2014), the results of the current study provide evidence for a specific inhibitory deficit in older adults. Theories that discussed a specific inhibitory deficit claimed that it is a general deficit in older adults (the *inhibitory deficit framework*; Hasher & Zacks, 1988; Hasher et al., 1991; Lustig et al., 2007). According to this theory, three main functions of inhibition are impaired in older adults: (a) access – preventing irrelevant information from gaining access to the focus of attention, (b) deletion – deleting irrelevant information from the focus of attention, and (c) restraint – suppressing the irrelevant response. Notably, this theory does not differentiate between controlling irrelevant information and controlling the irrelevant task, but refers to the general ability to inhibit irrelevant information. Researchers often interchangeably use inhibition of task and inhibition of information as referring to the same process (e.g., see Lustig et al., 2007, p. 154). Our results suggest that there is a more specific inhibitory deficit in older adults which is limited to controlling irrelevant information, while the ability to control irrelevant tasks is not compromised. This suggestion supports previous studies that refuted the generic inhibitory deficit idea by showing that other inhibitory functions remain intact. For example, Burca et al. (2022) found that older adults failed to inhibit the semantic information of the words but exhibited no problem in inhibiting the irrelevant response as compared to younger adults. In addition, Augustinova et al. (2018) found that the semantic Stroop effect (the difference in RT of the word SKY in yellow color and a neutral non-color related word), which represents the ability to inhibit irrelevant objects’ semantically related colors, remains intact in older adults. The results of the present study also agree with the initial evidence provided by Li and Bosman (1996), who found no age-related differences between neutral words and non-words. We suggest that the automatic activation of task conflict, which is something that is acquired at a very young age (Ben-Shalom et al., 2013; La Heij & Boelens, 2011; La Heij et al., 2010), seems to be stable across the life span. However, the ability to resolve information conflict, which requires greater flexibility, diminishes with age.

In addition to our primary analyses, we conducted supplementary analyses using Z-scored RTs to further investigate the potential dependency between task conflict and information conflict, following the approach of Jackson and Balota (2013). This additional analysis, detailed in the Online Supplementary Materials, suggests that both task conflict and information conflict are significant for both younger and older adults. However, only information conflict showed a significant age-related difference, while task conflict did not. These findings, which are in essence identical to the log-normal Bayesian analysis reported in the *Results* section, imply that the ability to maintain the task set over time may be relatively preserved in older adults, consistent with Jackson and Balota's findings. This supports the notion that age-related deficits might be more pronounced in inhibitory control mechanisms than in the ability to maintain the task set (Jackson & Balota, 2013; Jong, 2001; West & Baylis, 1998; West & Schwarb, 2006).

The results of the current study are also in accordance with the extensive meta-analysis by Rey-Mermet and Gade (2018), which included 176 studies that tested inhibition in older adults and showed no clear age-related inhibitory deficits, a finding that calls into question the hypothesis of the general inhibition deficit theory. Our findings specifically show that the irrelevant task of reading is inhibited in older adults to the same extent that it is inhibited in younger adults. However, it is not clear whether the lack of task control deficit is specifically related to the reading task or to a generally preserved ability to inhibit irrelevant tasks. Given the large evidence for preserved or even improved lexical abilities in older adults (Hardy et al., 2017; Salthouse, 2004), it is crucial to test whether this lack of deficit in inhibiting the irrelevant task of reading is related to the fact that the irrelevant task is a lexical task. Future studies, therefore, can use other irrelevant cognitive tasks to test task control in older adults as compared to younger adults.

The results of the current study can also be discussed in light of the dual mechanism of control framework (Braver, 2012; Braver et al., 2007). According to this theory, there are two, qualitatively different, cognitive control mechanisms: The first is proactive control, which is a capacity-demanding mechanism, in which goal-relevant information is actively maintained in a sustained manner. Proactive control acts in accordance with one’s internal goals prior to the appearance of the stimulus and is used to prevent or reduce interference. The second, reactive control, is a mechanism that acts after the stimulus onset to resolve the interference ad hoc. Most of the early studies that tested proactive control mechanisms across age groups have employed different manipulations to test whether the functionality of these mechanisms differs between older and younger adults (e.g., Manard et al., 2017; Mutter et al., 2005; West & Baylis, 1998). These studies that used the conflict-frequency manipulation with older adults found no age-related effects for the difference between the Stroop interferences under low proactive control (high frequency of congruent or neutral trials) versus high proactive control conditions (high frequency of incongruent trials), which suggests there are no differences in the efficiency of the proactive control between older and younger adults. This lack of an interaction between age and conflict-frequency on the Stroop interference effect was observed when researchers measured the interference effect as incongruent versus neutral words (e.g., West & Baylis, 1998), incongruent versus neutral symbols (Manard et al., 2017; Mutter et al., 2005), and as incongruent versus congruent trials (e.g., Bugg et al., 2008; Cohen-Shikora et al., 2018). Despite this consistent evidence from earlier studies, it has been suggested that proactive control might be compromised in older adults. In some other studies that utilized other tasks to test proactive and reactive control as a function of age, proactive control was found to be impaired in older adults (Paxton et al., 2008). In addition, Bugg (2014) suggested that the design of the studies mentioned above was contaminated, such that reactive control was also manipulated in those designs, and, indeed, in recent studies that employed more advanced designs to measure proactive and reactive control, older adults exhibited a decline in proactive control (Ball et al., 2023; Ilery-Tayar et al., 2024).

We believe that our results can provide another important piece of the age-related proactive control deficit puzzle. All the studies mentioned above that dealt with the question of whether proactive control is impaired in older adults, without exception, tested information conflict and often neglected markers that contained other processes (task conflict, facilitation, etc.). In the current study, we have shown that age-related differences in cognitive control in the Stroop task depend on the type of conflict that is measured and are not a result of reduced task control in older adults. Therefore, in future research, to be able to understand if and to what extent the proactive deficit in older adults exists, task conflict and the exertion of task control (which does not seem to be impaired in older adults) should not be neglected. Specifically, to fully determine whether there is no deficit in task control, we suggest testing task conflict under high control conditions, to be able to determine whether older adults struggle in exerting task control when task conflict is more frequent.

Another significant aspect of the current research is the type of task used to test differences in task conflict. In the current study, participants completed a manual task requiring keyboard responses, rather than a vocal task that uses a microphone to measure RT. Notably, task conflict measures are typically stronger in the vocal Stroop task (Augustinova et al., 2019; Kinoshita et al., 2018). This suggests that the vocal task, which measures a larger task conflict measurement, might reveal differences between older and younger adults that the manual task failed to observe. Additionally, vocal and manual tasks are expected to trigger different lexical processes (Keha & Kalanthroff, 2023b; Roelofs, 2003), which could influence age-related differences in task-conflict activation. To comprehensively determine whether task conflict activation is truly unimpaired in older adults, future studies should incorporate vocal tasks alongside manual ones or employ measures that do not rely on RTs and can provide a temporal resolution of the conflicts such as pupillometry or other neurological markers.

Finally, the current study results might have an important contribution to the broader task conflict literature. One crucial theoretical question is whether task and information control are truly two distinctive and independent mechanisms. Several studies have found evidence for the independence of these two conflict types. The first evidence came from Kalanthroff et al. (2013), who found a task conflict in the absence of information conflict trials. However, recent work by Entel and Tzelgov (2020) challenged these findings with a series of experiments showing that the reverse facilitation effect, a marker of task control, is only apparent in the presence of incongruent trials during practice. In another work, the question of dependency between conflict types was tested using a pupillometry technique (Hershman & Henik, 2020). This study found that the task conflict emerged in an early stage of the trials (about 500 ms after stimulus onset) but information conflict emerged only in a later stage (about 1,000 ms after stimulus onset). Similar conclusions were drawn by Steinhauser and Hübner (2009), who analyzed the RT distribution of the Stroop task. In the current study, an age-related dissociation between the emergence of task and information conflict was found, a result that supports the conflict-type independence hypothesis. Note that other studies that tested differences in conflict types between different populations failed to observe this dissociation. However, even though we used the most common theoretical framework in the literature – defining task conflict as the difference between the neutral word condition and the neutral symbol condition or by the reverse facilitation effect, and information conflict as the difference between the incongruent color condition and the neutral word condition – there remains a question of whether these constructs are truly independent (Kalanthroff et al., 2013; Parris et al., 2023). Therefore, future studies that will explore the true nature of dependency between these two constructs will allow us to further understand how these two separate mechanisms act in older adults compared to the younger group.

In summary, our results revealed, for the first time, that there is a dissociation between task control and information control in older adults. Using the Stroop color-word task under low control conditions, we found that as compared to younger adults, older adults did not exhibit any problems in inhibiting the irrelevant task of word reading but did exhibit a deficit in inhibiting irrelevant information when the word and color were incongruent. These results challenge the global inhibition deficit model for older adults' cognitive functioning and suggest that a more specific inhibition deficit model is warranted. The current study results have important implications for our understanding of cognitive processes during aging as they demonstrate that the cognitive deficit in conflict resolution is more specific than previously thought.

## Electronic supplementary material

Below is the link to the electronic supplementary material.

Supplementary file1 (DOCX 28 KB)

## Data Availability

The data supporting the findings of this study are openly available on the Open Science Framework (OSF) at https://osf.io/a9dmh/.

## Appendix A

We fit a Bayesian log-normal linear model to predict Stroop reaction time (RT), which included one between-subject variable: group (older adults vs. younger adults), one within-subject variable: stimulus type (congruent, symbols, words, and incongruent), and the interaction effects between these predictors, with orthonormal coding. Accordingly, the between-subject variables were inserted into the model as fixed effects, whilst stimulus type was both a fixed effect and random slope for the subjects – the random variable. Additionally, we modeled the scale component of the RT’s distributions, but we did not analyze them in this paper. Also, we allowed the correlation between the random intercept and slopes (between participants) of the RTs and scale for a better model fit.

The model was fit using the brms package in R (Bürkner, 2017), and further analyses were conducted using the emmeans (Lenth et al., 2018) and bayestestR (Makowski et al., 2019) packages. The model’s syntax was as follows:

$$\begin{array}{l}\text{brm}(\\ \text{formula }=\text{ bf}(\text{RT }|\text{ trunc}(\text{ub }= 2) \sim \text{ Type }*\text{ Group }+ (\text{Type }|\text{ p }|\text{ Subject}),\\ \begin{array}{l}\text{sigma }\sim 1 + (1 |\text{ p }|\text{ Subject})),\\ \text{family }=\text{ lognormal}(\text{Link function}:\text{ identity}),\\ \begin{array}{l}\text{iter }= 4000,\text{warmup }= 2000,\\ \text{chains }= 4,\\ \begin{array}{l}\text{control }=\text{ list}(\text{adapt}\_\text{delta }= 0.90,\\ \text{ max}\_\text{treedepth}=12))\end{array}\end{array}\end{array}\end{array}$$

For the priors, we used conservative priors for the coefficients with normal distributions centered on zero and, where it was possible, we used theoretically informed priors. Thus, for the age-related differences in the Stroop interference, we set a normal distribution with SD = 1.52, allowing a 50% chance of this effect to vary between ± 291 ms, which is a reasonable difference between Stroop interference of younger and older adults based on previous studies (Verhaeghen & De Meersman, 1998). In a similar vein, based on previous studies that tested task conflict in the Stroop task (Augustinova et al., 2018, 2019; Keha & Kalanthroff, 2023a, Kinoshita et al., 2018; Kinoshita et al., 2017; McClain, 1983; Redding & Gerjets, 1977; Zahedi et al., 2019), a normal distribution with SD = 1.97 was set for the difference between the age groups allowing a 50% chance of this effect, to vary between ± 30 ms. For the other coefficients, a weakly generic informative normal distribution with SD = 1 was employed.

**Left panel:** Examples of MCMC chains for some of the model parameters. **Right panel:** Posterior predictive check of the model.

## Footnotes

1 For the full list of stimuli, presented colors, and their proportions, see Table 1 in the Online Supplementary Material (OSM) A.

2 In addition to the main analysis that was conducted, we also applied a Bayes factor analysis to account for the null age-related effect in task conflict. For a detailed discussion of our Bayesian inference approach and methodological considerations, see OSM C.

## Declarations

### Open Practices Statement

The study design and analytic plan were not pre-registered. The full model and the relevant data for this work can be all found via the Open Science Framework at: https://osf.io/a9dmh/?view_only=b4e1ce814bbf48a89552b1e5faa335fe. The analytic code needed to reproduce analyses is given in Appendix A.
