# Supplementary material for: Age-related differences in information, but not task control in the color-word Stroop task
Source: Psychon Bull Rev. 2025 Jan 17;32(4):1551–61. doi: 10.3758/s13423-024-02631-z (PMC12325456; doi:10.3758/s13423-024-02631-z)
Supplement: Supplementary file 1 — Supplementary file1 (DOCX 28 KB) [file 13423_2024_2631_MOESM1_ESM.docx]

**Supplementary**

1. **Table of Stimuli and their Proportions**

**Table 1**

|  | **Neutral Symbols** | **Congruent** | **Incongruent** | **Neutral Words** | **Presented Color** |
| --- | --- | --- | --- | --- | --- |
|  | **+=!/** | צהוב (YELLOW( | ) ירוקGREEN( | ) תנורOven( | Yellow |
|  | ***?!#** | ) ירוקGREEN( | ) כחולBLUE( | ) דוכןStall( | Green |
|  | **%$!@** | ) אדוםRED( | צהוב (YELLOW( | ) טופסForm( | Red |
|  | **~>!"** | ) כחולBLUE( | ) אדוםRED( | ) סירPot( | Blue |
| **Condition’s Proportion** | 66.7% (16.67% for each stimulus) | 11.11% (2.78% for each stimulus) | 11.11% (2.78% for each stimulus) | 11.11% (2.78% for each stimulus) |  |

*Note.* Supplementary Table 1 presents the proportions and characteristics of stimuli used in the study as well as the presented color of each of the stimuli in the experiment. Note that each stimulus in the experiment appeared only in one color. All the words in the experiment appeared in Hebrew and the English translation of each word in the Table is presented in parentheses.

1. **Traditional Analysis of Variance**

Based on the methodology outlined by Jackson and Balota (2013), we conducted a z-score transformation on the within-subject data to normalize the reaction times (RTs; see Table 2). A 4X2 mixed model analysis of variance (ANOVA) was carried out on accuracy rates with stimulus-type (congruent vs. neutral words vs. incongruent vs. neutral symbols) as a within-subject factor and age group as a between-subject factor (older adults vs younger adults). Significant main effects were found for age group, *F*(1, 53) = 8.64, *p* = .005, η²_p_ = 0.140, with older adults slower than younger adults and for stimulus-type, *F*(3, 159) = 95.60, *p* < .001, η²_p_ = 0.643. Post-hoc analysis using a Bonferroni correction revealed that neutral symbols were significantly different than congruent, incongruent and neutral words (all p’s were <.001) and that incongruent trials were significantly different than neutral words and congruent trials (both p’s were <.001) but words and congruent trials did not differ (p = .99). Finally, a significant interaction between stimulus-type and age group was found, *F*(3, 159) = 17.64, *p* < .001, η²_p_ = 0.250.

To further analyze this 2-way interaction, we computed the age-related differences between younger and older adults in task conflict (neutral words – symbols), information conflict (incongruent - neutral words), reverse facilitation effect (symbols – congruent) and regular facilitation effect (words - congruent). We first tested each the significance of each possible comparison separately for older and younger adults using a Bonferroni correction to account for the usage of multiple comparisons and type 1 error increase. The descriptive and inferential statistics of each contrast in each of the two groups are presented in Table 3. In addition, we tested whether the main contrasts of interest differ between younger and older adults. The results showed that only in the information conflict marker, *t*(53) = 4.363, p < .001, Cohen’s d = 1.20, but not for the task conflict, *t*(53) = 0.193, p = .847, reverse facilitation, *t*(53) = 1.314, p = .194, nor the regular facilitation, *t*(53) = 1.327, p = .190.

**Table 2**

|  | Congruent | Neutral words | Incongruent | Neutral symbols | **Group Mean** |
| --- | --- | --- | --- | --- | --- |
| Older Adults | -0.022 (0.041) | 0.007 (0.033) | 1.004 (0.086) | -0.146 (0.015) | 0.211 (0.021) |
| Younger Adults | 0.117 (0.042) | 0.043 (0.034) | 0.437 (0.087) | -0.099 (0.015) | 0.125 (0.021) |
| **Condition Mean** | 0.047 (0.029) | 0.026 (0.024) | 0.721 (0.061) | -0.122 (0.010) |  |

*Note.* Means (SE) data of each condition in each age group.

**Table 3**

**Pairwise Comparisons of Stimulus-Type within each Age Group**

| Older Adults | Comparison | Estimate | Standard Error | df | t-score | p-value |
| --- | --- | --- | --- | --- | --- | --- |
|  | Neutral Symbols - Incongruent | -1.1496 | 0.0978 | 53 | -11.757 | <.0001 |
|  | Neutral Symbols - Neutral Words | -0.1534 | 0.0392 | 53 | -3.910 | 0.0016 |
|  | Neutral Symbols - Congruent | -0.1240 | 0.0489 | 53 | -2.536 | 0.0851 |
|  | Incongruent - Neutral Words | 0.9962 | 0.0967 | 53 | 10.303 | <.0001 |
|  | Incongruent - Congruent | 1.0256 | 0.0955 | 53 | 10.735 | <.0001 |
|  | Neutral Words - Congruent | 0.0294 | 0.0541 | 53 | 0.543 | 0.9999 |
| Younger Adults | Neutral Symbols - Incongruent | -0.5367 | 0.0996 | 53 | -5.390 | <.0001 |
|  | Neutral Symbols - Neutral Words | -0.1425 | 0.0399 | 53 | -3.569 | 0.0046 |
|  | Neutral Symbols - Congruent | -0.2157 | 0.0498 | 53 | -4.332 | 0.0004 |
|  | Incongruent - Neutral Words | 0.3942 | 0.0985 | 53 | 4.003 | 0.0012 |
|  | Incongruent - Congruent | 0.3210 | 0.0973 | 53 | 3.300 | 0.0104 |
|  | Neutral Words - Congruent | -0.0731 | 0.0551 | 53 | -1.327 | 0.9999 |

1. **Bayesian Inference Analysis and Relevant Considerations**

While our main analysis presents posterior distributions, we also conducted supplementary analyses using Bayes factors to compare effect sizes of task conflict between age groups. These analyses yielded BF01>3, supporting our finding of no significant age-related differences. However, two important methodological considerations warrant mention. First, Bayes factors are highly sensitive to prior specification (Makowski et al., 2019), which is particularly challenging in our field given limited prior knowledge of age-related differences in task conflict. Our analysis relied on generic weakly informative priors, necessitating cautious interpretation. Alternative approaches like the Region of Practical Equivalence (ROPE) could potentially address effect size comparisons in a Bayesian framework. However, determining appropriate ROPE boundaries would require stronger empirical foundations from future research. Our approach of presenting full posterior distributions provides comprehensive information about both effect magnitudes and uncertainty, allowing readers to evaluate the substantial overlap between age groups in task conflict and reverse facilitation effects. Our open data and analysis code enable future researchers to apply alternative parameterizations or priors as the field develops.

**References**

Makowski, D., Ben-Shachar, M. S., Chen, S. A., & Lüdecke, D. (2019). Indices of effect existence and significance in the Bayesian framework. *Frontiers in Psychology*, *10*, 2767.
